# Feasibility of robotic liver resection compared with laparoscopic and open liver resection for hepatocellular carcinoma: a network meta-analysis

**DOI:** 10.1097/JS9.0000000000003528

**Published:** 2025-10-09

**Authors:** Sang-Hoon Kim, Ki-Hun Kim, Hugo Pinto-Marques, João Santos Coelho

**Affiliations:** a Division of Liver Transplantation and Hepatobiliary Surgery, Department of Surgery, Asan Medical Center, University of Ulsan College of Medicine, Seoul, Korea; b NOVA Medical School, Hepato-Biliary-Pancreatic and Transplantation Centre Curry Cabral Hospital/Local Health Unit of São José, Lisbon, Portugal

**Keywords:** hepatocellular carcinoma, laparoscopic liver resection, network meta-analysis, open liver resection, robotic liver resection

## Abstract

**Background:**

Robotic liver resection (RLR) has gained popularity in the treatment of hepatocellular carcinoma (HCC); however, its efficacy compared to laparoscopic liver resection (LLR) and open liver resection (OLR) remains unclear.

**Methods:**

Comprehensive literature search of electronic databases from January 2010 to December 2024 identified studies comparing RLR, LLR, or OLR. Operative, postoperative, and survival data were extracted, and pooled odd ratios or hazard ratios with 95% confidence intervals were calculated using a frequentist network meta-analysis including RLR, LLR, and OLR.

**Results:**

A total of 69 studies, comprising 1 randomized controlled, 3 prospective, and 65 retrospective-matched studies, involving 13,257 patients were analyzed. This network meta-analysis showed that RLR had significantly lower blood loss than both OLR and LLR, with comparable operative time, RBC transfusion rates, Pringle maneuver use, Pringle time, and R1 resection rates. RLR showed similar rates of overall and major complications and hospital stay duration as LLR, with significant benefits over OLR. No significant differences in 90-day mortality were found among the three groups. For long-term outcomes, RLR showed no significant advantage over LLR or OLR in overall and recurrence-free survival, though it generally ranked higher with a greater *P*-score.

**Conclusions:**

This network meta-analysis suggests that RLR is a feasible surgical treatment option for HCC, offering perioperative and long-term outcomes comparable to LLR, with reduced postoperative morbidity and shorter hospital stays compared to OLR. However, further studies are needed to confirm RLR’s efficacy due to its limited sample size.

## Introduction

Hepatocellular carcinoma (HCC) remains a major global health challenge, ranking as the fourth leading cause of cancer-related death. While open liver resection (OLR) has traditionally been considered the gold standard for curative treatment of HCC, minimally invasive liver surgery, including laparoscopic and robotic techniques, has recently become more common due to its advantages over OLR^[1,2]^. Laparoscopic liver resection (LRL) has become widely adopted globally^[2]^, but laparoscopic procedures face several limitations, such as restricted instrument movement, two-dimensional visualization, instability, suboptimal ergonomics, and reliance on assistants, which can complicate its use, particularly given the inherent complexity and variability of liver surgeries^[3]^. The advent of robotic liver resection (RLR) offers a solution to some of these challenges by providing a broader range of motion, enhanced three-dimensional visualization, and improved stability and ergonomic conditions, potentially enhancing surgical precision and outcomes^[4]^. Consequently, minimally invasive liver surgery has shifted from laparoscopic toward robotic platform over the past decade^[5]^.

**HIGHLIGHTS**

- This network meta-analysis compares robotic, laparoscopic, and open liver resection.
- Robotic liver resection had less blood loss than laparoscopic and open surgery.
- Robotic liver resection had similar complications and shorter hospital stays.
- Overall and recurrence-free survival were similar across all three approaches.
- Robotic liver resection is a safe treatment for hepatocellular carcinoma patients.

Numerous previous studies and meta-analyses have demonstrated the advantages of LLR over OLR for surgical treatment of HCC, including reduced blood loss, shorter hospital stays, and a lower incidence of postoperative complications^[6–8]^. Despite rapid advancements in RLR for HCC, high-quality studies directly comparing RLR with LLR or OLR remain limited, particularly regarding long-term outcome^[9]^. Most comparative studies on RLR have primarily focused on short-term outcomes from various tumors, with minimal data available on long-term effects specific to HCC^[10–15]^. Consequently, comprehensive meta-analyses evaluating both short- and long-term impacts of RLR versus LLR or OLR in HCC patients are lacking. Several previous meta-analyses have reported the perioperative advantages of minimally invasive liver surgery, including RLR and LLR, over OLR; however, it is still inconclusive whether robotic, laparoscopic, or open surgical approaches provide better long-term survival benefits for HCC patients^[12,13,16]^. Therefore, a comprehensive analysis incorporating recent evidence to compare RLR, LLR, and OLR for HCC is essential to clarify their perioperative and long-term outcomes.

This study aimed to assess the impact of RLR on both short- and long-term outcomes in HCC patients compared to LLR and OLR through a network meta-analysis. Due to the limited availability of high-quality studies directly comparing RLR and LLR for HCC, we employed an indirect approach by pooling data from well-designed studies comparing RLR with LLR, LLR with OLR, and RLR with OLR. This method allowed us to maximize the sample size related to RLR and LLR, thereby providing conclusions with greater reliability despite the limited number of direct comparative studies.

## Methods

This study was reported in accordance with the TITAN criteria, Transparency in the Reporting of Artificial Intelligence, as summarized in Supplemental Digital Content Table S1, available at http://links.lww.com/JS9/F301^[17]^.

### Data collection and search strategy

This systematic review and network meta-analysis followed the guideline of the Cochrane Handbook for Systematic Reviews of Interventions^[18]^, PRISMA (Preferred Reporting Items for Systematic Reviews and Meta-Analyses)^[19]^, and AMSTAR 2 (Assessing the methodological quality of systematic reviews 2)^[20]^. Institutional Review Board approval and patient consent were not required, since this study based entirely on published data. Comprehensive literature search was conducted using multiple databases, including Embase, Cochrane Library, Web of Science, and PubMed, complemented by manual searches covering publications from January 2010 to October 2024. The search strategies are outlined in Supplemental Digital Content Table S2, available at: http://links.lww.com/JS9/F301. The protocol for the study was registered in Research Registry (Unique Identifying Number: review registry 1915).

### Study selection

Two investigators independently conducted the search to identify articles for inclusion in this meta-analysis. Eligibility disagreements were resolved through discussion and consensus. The inclusion criteria for this study are follows: (1) studies including patients diagnosed with primary HCC; (2) studies reporting outcomes including operative, postoperative, or long-term outcomes, such as overall survival (OS) or recurrence-free survival (RFS), and (3) full-text original articles published in English. Exclusion criteria were as follows: (1) studies involving patients who underwent hepatectomy for reasons other than HCC; (2) case reports, non-comparative studies, duplicates, and reviews; and (3) unpublished studies.

### Data extraction

Two independent researchers extracted data from the selected studies. The information collected included publication year, country, study design, study period, sample size, patient demographics (age and sex), Child-Pugh classification, cirrhosis status and etiology, tumor characteristics (size and number), and preoperative alpha-fetoprotein levels. Long-term survival data, including hazard ratios (HRs) for OS and RFS at 1-, 3-, and 5-year intervals, were also extracted. The primary outcomes were long-term outcomes including OS and RFS. Furthermore, secondary outcomes comprised operative and postoperative outcomes, including operative time, estimated blood loss, red blood cell transfusion, Pringle maneuver, Pringle time, R1 resection, overall and major complication, 90-days mortality, and hospital stays.

### Quality assessment of studies

The quality of randomized trials was evaluated using the Cochrane risk-of-bias tool for randomized trials, version 2, which categorizes bias risk as “low risk,” “some concerns,” or “high risk” based on responses to specific signaling questions^[21]^. For retrospective and prospective studies, we applied the Newcastle–Ottawa Scale, assigning scores from 0 to 9, with scores of 7 or above considered “high quality”^[22]^. Quality assessment of all included studies was independently conducted by two reviewers, who resolved any discrepancies through discussion and consensus.

### Statistical analysis

Dichotomous variables were analyzed using odds ratios (ORs) with 95% confidence intervals (CIs), while continuous variables were assessed through standardized mean differences (SMDs) with 95% CIs. We used the method proposed by Wan to estimate the sample mean and standard deviation from the sample size, median, range, and/or interquartile range^[23]^. Survival outcomes – including 1-, 3-, and 5-year OS and RFS – were presented as pooled data, with HRs calculated and compared across different time points. HRs for OS and RFS were reconstructed from time-to-event data extracted from Kaplan–Meier curves, following the method outlined by Tierney *et al*^[24]^. Engauge Digitizer 4.1 (http://digitizer.sourceforge.net), a tool for analyzing graphical images and extracting data points from graphs, was used to capture time-to-event data from the Kaplan–Meier curves. Meta-analyses incorporating two-arm studies comparing RLR, LLR, and OLR were conducted. In addition, network meta-analyses were conducted within the frequentist framework, using *P*-scores to rank the effectiveness of different surgical approaches^[25]^. To address the heterogeneity among studies – such as differences in surgical techniques, surgeon expertise, patient demographics, geographic locations, patient management, and study durations – a random effects-model was deemed appropriate over a fixed effects model^[26]^. Sensitivity analyses were performed by excluding studies identified as non-high-quality. Publication bias was evaluated through a comparison-adjusted funnel plot^[27]^. Statistical significance was set at *P* < 0.05. All statistical analyses were conducted using Review Manager 5.4 (Cochrane Collaboration, Oxford, UK) and R version 4.4.1 (https://cran.r-project.org).

## Results

### Literature search

**Figure 1.**
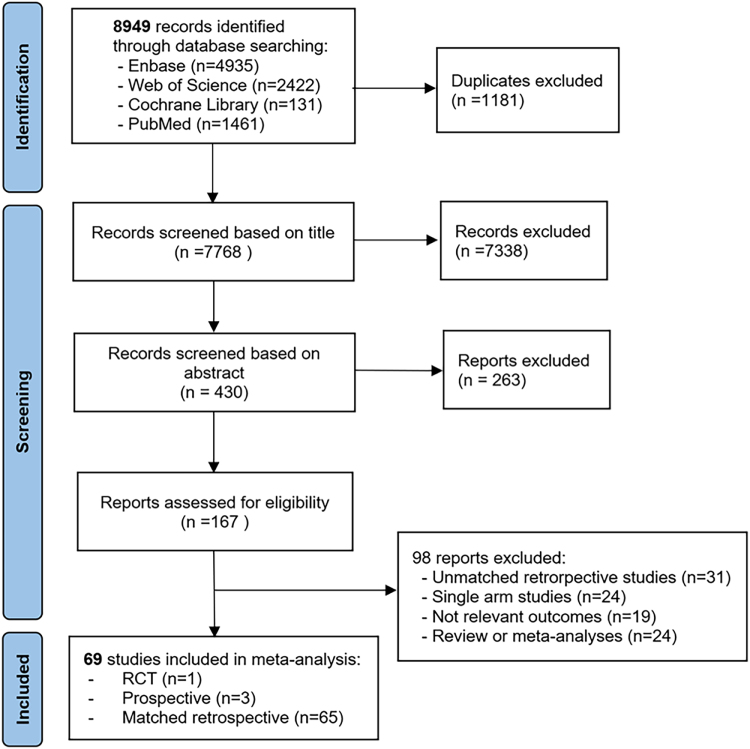
PRISMA flowchart of study inclusion.

A total of 8949 studies were initially identified through electronic database searches. Following the removal of duplicates and a title and abstract screening, 167 articles were deemed eligible for further review. Among these, 98 articles were subsequently excluded for various reasons, such as not meeting the inclusion criteria, single-arm studies, unmatched cohort studies, or lacking original data. Finally, 69 studies published between 2010 and 2024 were incorporated into this meta-analysis, including 1 randomized controlled trial (RCT)^[28]^, 3 prospective^[1,29,30]^, and 65 retrospective-matched studies^[9,31–94]^. The study selection is summarized in the PRISMA flowchart (Fig. 1).

### Study characteristics

Table 1 summarizes the baseline characteristics of the 69 studies included in this meta-analysis, comprising 13 257 patients.

**Table 1:** Characteristics of included studies

Study	Region	Design	Study period	Study arm	*N*	Age (year)	Sex (M/F)	BMI (kg/m^2^)	CTP (A/B/C)	Cirrhosis (%)	HBV (%)	Tumor size (cm)	AFP (ng/mg)	Solitary tumor	NOS/ROB
Laparoscopic vs. robot (656 vs. 263, Total *N* = 919)															
Duong 2022^[49]^	USA	RM	2010–2015	LLR	369	62 (57–69)	268/101	–	–	–	–	2.7 (2.0–3.5)	–	–	7
				RLR	123	62 (55–70)	88/35	–	–	–	–	2.8 (2.2–3.6)	–	–	
Li 2024^[35]^	China	RM	2016–2021	LLR	244	53.7 ± 10.1	202/42	24.3 (22.5–26.9)	–	202 (82.8)	220 (90.2)	3.1 (2–5)	13 (4–235)	229 (94)	9
				RLR	97	53.0 ± 11.09	78/19	25.0 (22.5–26.8)	–	81 (83.5)	84 (86.6)	3.5 (2–4.7)	14 (4–274)	89 (92)	
Huang 2024^[9]^	China	RM	2017–2023	LLR	43	>60: 19 (44.2)	35/8	–	41/2/–	32 (74.4)	33 (76.7)	–	>20: 17 (39.5)	43 (100)	8
				RLR	43	>60: 23 (53.5)	35/8	–	40/3/–	32 (74.4)	34 (79.1)	–	>20: 18 (41.9)	43 (100)	
Open vs. robot (843 vs. 580, Total *N* = 1423)															
Chen 2017^[75]^	Taiwan	RM	2012–2015	OLR	81	–	–	–	–	37 (45.7)	–	–	–	–	7
				RLR	81	–	–	–	–	38 (46.9)	–	–	–	–	
Zhang 2022^[50]^	China	RM	2010–2020	OLR	178	>75: 35 (19.7)	144/34	–	155/23/–	124 (69.7)	149 (83.7)	>5: 69 (38.8)	>400: 48 (27)	155 (87)	8
				RLR	100	>75: 19 (19)	78/22	–	85/15/–	70 (70.0)	82 (82.0)	>5: 39 (39)	>400: 26 (26)	83 (83)	
Sucandy 2022^[47]^	USA	RM	2016–2021	OLR	13	–	–	–	–	–	–	–	–	–	7
				RLR	13	–	–	–	–	–	–	–	–	–	
Benedetto 2023^[37]^	Italy	RM	2010–2020	OLR	106	69 (63–72)	88/18	26.9 (22.2–29.6)	100/6/–	–	–	3 (2.1–4.5)	6 (3–25)	83 (78.3)	9
				RLR	106	67 (59–72)	85/21	25.8 (23.7–29.0)	101/5/–	–	–	3.5 (2.3–5)	35 (23–50)	95 (89.6)	
Zhang 2024^[36]^	China	RM	2010–2020	OLR	465	>60: 149 (32)	385/80	24.2 (22.6–26.2)	–	293 (63)	–	>10: 89 (19)	>400: 170 (37)	407 (88)	8
				RLR	280	>60: 108 (39)	232/48	24.3 (22.5–25.9)	–	186 (66)	–	>10: 48 (17)	>400: 98 (35)	247 (88)	
Open vs. laparoscopic vs. robot (122 vs. 82 vs. 70, Total *N* = 274)															
Zhu 2023^[1]^	China	P	2015–2016	OLR	56	53 (21–73)	44/12	23.1 (16.6–28.4)	49/7/–	–	43 (76.8)	4.0 (1.2–14)	23 (2–60 500)	54 (96.4)	7
				LLR	56	53 (24–72)	47/9	23.3 (16.6–31.2)	49/7/–	–	48 (85.7)	3.3 (1.1–14.3)	28 (1–8753)	53 (94.6)	
				RLR	56	52 (28–72)	45/11	23.4 (15.9–30.9)	50/6/–	–	49 (87.5)	3.3 (1.0–12.5)	43 (3–60 500)	52 (92.9)	
O’Connell 2023^[43]^	Ireland	RM	2012–2022	OLR	66	62.8 ± 14.7	50/16	–	–	32 (48)	–	5 (1.3–19.5)	–	59 (89)	9
				LLR	26	65.4 ± 10.7	23/3	–	–	12 (46)	–	4.3 (0.7–17.5)	–	25 (96)	
				RLR	14	64.4 ± 7.0	9/5	–	–	12 (86)	–	4.3 (1.2–9.2)	–	14 (100)	
Open vs. laparoscopic (6199 vs. 4442, Total *N* = 10 641)															
Aldrighetti 2010^[93]^	Italy	RM	2005–2009	LLR	16	65 ± 10	11/5	–	9/–/–	9 (56.3)	–	4.0 ± 2.2	–	–	7
				OLR	16	71 ± 6	12/4	–	9/–/–	9 (56.3)	–	4.6 ± 2.0	–	–	
Tranchart 2010^[94]^	France	RM	2002–2009	LLR	42	63.7 ± 13.1	27/15	–	31/1/11	31 (73.8)	–	3.6 ± 1.8	–	–	7
				OLR	42	65.7 ± 7.1	28/14	–	33/1/8	34 (80.9)	–	3.7 ± 2.1	–	–	
Kim 2011^[92]^	Korea	RM	2005–2009	LLR	26	57.8 ± 9.6	18/8	23.8 ± 2.6	–	24 (92.3)	16 (61.5)	3.2 (1–8)	3.3 ± 2.3	–	6
				OLR	29	57.1 ± 9.8	20/9	22.9 ± 2.45	–	25 (86.2)	20 (69.0)	3.6 (1–19)	4.2 ± 2.8	–	
Cheung 2013^[91]^	China	RM	2002–2009	LLR	32	60 (39–79)	22/10	–	32/0/0	–	26 (81.3)	2.5 (1–10)	–	–	7
				OLR	64	61 (29–82)	50/14	–	60/4/0	–	49 (76.6)	3 (1–10)	–	–	
Memeo 2014^[90]^	France	RM	1990–2009	LLR	45	62 (34–75)	35/10	–	44/1/–	–	16 (35)	3.2 (0.9–11)	11 (1–2400)	–	7
				OLR	45	60 (43–80)	37/8	–	43/2/–	–	13 (29)	3.7 (0.1–15)	13 (1–5200)	–	
Ahn 2014^[89]^	Korea	RM	2005–2013	LLR	51	58.2 ± 10.4	36/15	23.8 ± 2.6	51/–/–	35 (68.6)	40 (78.4)	2.6 ± 1.5	504.0 ± 986.1	–	8
				OLR	51	57.1 ± 10.6	40/11	24.1 ± 3.4	51/–/–	34 (66.8)	37 (72.5)	2.8 ± 1.2	262.6 ± 586.2	–	
Kim 2014^[88]^	Korea	RM	2000–2012	LLR	29	54.6 ± 9.2	22/7	23.9 ± 2.6	28/1/–	18 (62.1)	–	–	1054 ± 4114	24 (82.8)	8
				OLR	29	53.9 ± 10.1	19/10	22.4 ± 4.7	29/–/–	19 (65.5)	–	–	4077 ± 10 683	28 (96.6)	
Luo 2015^[85]^	China	RM	2008–2015	LLR	53	49 (36–72)	38/15	–	–	–	41 (77.4)	3 (2–5)	–	–	7
				OLR	53	51 (38–68)	35/18	–	–	–	38 (71.7)	3 (1–6)	–	–	
Tanaka 2015^[86]^	Japan	RM	2007–2014	LLR	20	70 (66–73)	17/3	–	–	–	4 (20)	2.3 (2.0–2.7)	–	18 (90)	8
				OLR	20	71 (67–75)	14/6	–	–	–	2 (10)	2.3 (1.9–2.8)	–	17 (85)	
Han 2015^[84]^	Korea	RM	2004–2013	LLR	88	60 (26–81)	72/16	23.7 (15.9–35.4)	79/6/3	55 (62.5)	61(69.3)	3 (1–12)	14 (1–35 000)	67 (76.1)	9
				OLR	88	60 (20–85)	74/14	23.6 (15.7–33.8)	77/9/2	52 (59.0)	65 (87.5)	3 (1.5–15)	11 (1–34 100)	70 (79.5)	
Yoon 2015^[82]^	Korea	RM	2007–2011	LLR	58	54 (49–63)	45/13	–	53/5/–	–	54 (93.1)	2.9(0.7–4.9)	2553 (3–132)	58 (100)	7
				OLR	174	55 (49–61)	130/44	–	158/16/–	–	165 (94.8)	3.0 (0.2–4.9)	469 (3–200)	174 (100)	
Takahara 2015^[83]^	Japan	RM	2000–2010	LLR	387	66.4 ± 9.8	262/125	–	312/65/10	–	91 (23.5)	2.9 ± 1.5	9.3 (4–62)	–	8
				OLR	387	66.2 ± 10.0	261/126	–	311/70/6	–	100 (25.8)	2.9 ± 1.5	13.5 (5–100)	–	
Lee 2015^[87]^	Canada	RM	2006–2013	LLR	43	62 (30–86)	29/14	–	41/1/–	–	19 (44.2)	5.4 (2–16)	–	41 (95.3)	7
				OLR	86	63 (34–84)	69/17	–	81/2/–	–	52 (60.5)	4.4 (2–14)	–	81 (94.2)	
Jiang 2015^[81]^	China	RM	2008–2013	LLR	59	51 (36–68)	42/17	20 (17–25)	–	–	35 (59.3)	3 (2–5)	–	–	7
				OLR	59	50 (38–70)	38/21	22 (18–28)	–	–	32 (54.2)	3 (1–6)	–	–	
Komatsu 2015^[79]^	France	RM	2006–2014	LLR	38	61.5 ± 12.2	34/4	25.2 ± 3.8	38/–/–	–	10 (26.3)	4.8 (2.3–18)	–	19 (50)	7
				OLR	38	61.7 ± 6.1	33/5	24.2 ± 4.0	38/–/–	–	9 (23.7)	8.5 (2.0–18)	–	16 (42.1)	
Sposito 2016^[78]^	Italy	RM	2006–2013	LLR	43	66 (40–85)	28/15	26.7 (16.6–39.8)	–	43 (100)	6 (14)	–	7 (0.3–19 454)	37 (86)	9
				OLR	43	68 (49–83)	35/8	26.7 (17.8–38.8)	–	43 (100)	10 (23)	–	6 (0.3–589)	35 (81)	
Cheung 2016^[80]^	China	RM	2004–2014	LLR	110	60 (32–84)	80/30	23.4 (12.9–35.7)	110/–/–	78 (70.9)	88 (80)	2.6 (0.6–10)	34 (2–8703)	100 (91)	7
				OLR	330	61 (25–89)	258/72	23.4 (15.3–35.2)	330/–/–	250 (75.8)	285 (86.4)	2.9 (0.8–10)	18 (1–54 860)	292 (89)	
Xiang 2016^[30]^	China	P	2012–2015	LLR	128	20.9 ± 11.9	109/19	–	108/20/–	104 (81.3)	106 (82.8)	6.7 ± 1.5	–	128 (100)	7
				OLR	207	55.5 ± 10.7	171/36	–	183/24/–	167 (80.7)	172 (83.1)	6.9 ± 1.5	–	207 (100)	
Wang 2017^[77]^	China	RM	2007–2015	LLR	184	56 (39–75)	128/56	22 (18–27)	184/–/–	–	168 (91.3)	–	–	–	7
				OLR	184	55 (39–74)	120/64	23 (17–29)	184/–/–	–	160 (87.0)	–	–	–	
Yoon 2017^[76]^	Korea	RM	2008–2015	LLR	33	56.0 ± 7.0	23/10	–	–	–	29 (87.88)	3.3 ± 1.7	–	–	8
				OLR	33	57.3 ± 6.9	26/7	–	–	–	28 (80.7)	3.0 ± 1.5	–	–	
Iwata 2018^[73]^	Japan	RM	2000–2013	LLR	30	70 (19–86)	18/12	–	26/4/-	–	7 (23.3)	–	48 (3–10 181)	29 (96.7)	9
				OLR	30	69 (28–82)	21/9	–	27/3/–	–	6 (20.0)	–	25 (2–4536)	28 (93.3)	
Xu 2018^[71]^	China	RM	2015–2017	LLR	32	54 (26–70)	28/4	22.8 (17.6–29.3)	–	–	18 (56.3)	4 (1–10)	11 (2–1210)	29 (90.6)	8
				OLR	32	52 (27–74)	28/4	22.2 (18.2–27.7)	–	–	15 (46.9)	6.2 (1.5–10)	56 (2–1210)	29 (90.6)	
Elgendi 2018^[28]^	Egypt	RCT	–	LLR	25	54.5 ± 7.0	16/9	29.0 ± 1.8	13/12/–	25 (100)	–	3.3 ± 0.6	230 ± 9	–	SC
				OLR	25	54.2 ± 7.4	14/11	28.0 ± 2.0	12/13/–	25 (100)	–	3.4 ± 0.6	226 ± 12		
Rhu 2018^[72]^	Korea	RM	2009–2016	LLR	53	58.0 ± 8.8	43/10	–	–	–	–	3.1 ± 1.8	6.6 ± 190.4	47 (88.7)	8
				OLR	97	58.2 ± 9.4	81/16	–	–	–	–	3.1 ± 1.7	8.1 ± 59.5	89 (91.8	
Kim 2018^[70]^	Korea	RM	2012–2016	LLR	37	58 (34–78)	30/7	–	–	15 (41.7)	27 (73.0)	2.8 (0.9–11.5)	13 (1–19 481)	–	8
				OLR	37	55 (29–79)	31/6	–	–	20 (54.1)	31 (83.8)	2.8 (1.1–10)	14 (1.3–14 841)	–	
Deng 2018^[74]^	China	RM	2002–2016	LLR	157	60 (31–85)	65/95	23.6 (17.2–34.5)	157/–/–	89 (56.7)	140 (89.2)	2.5 (0.8–10)	36 (3–8712)	138 (87.9)	8
				OLR	157	61 (26–85)	60/97	23.4(16.2–34.2)	157/–/–	92 (58.6)	143 (91)	2.9 (1–18)	34 (2–7820)	135 (86.0)	
Wu 2019^[69]^	China	RM	2010–2015	LLR	86	53 (17–79)	72/14	23.7 (15.3–33.8)	–	65 (75.6)	77 (89.5)	3.5 (0.9–12.5)	53 (69–856)	64 (74.4)	8
				OLR	86	52 (21–82)	74/12	23.6 (15.7–34.5)	–	62 (72.1%)	79 (91.9)	3.5 (0.8–11.3)	86 (12–1026)	58 (67.4)	
Fu 2019^[67]^	China	RM	2013–2015	LLR	14	61.5 (28–77)	10/4	–	–	8 (57.14)	–	6.0 (5.5–10.0)	–	–	8
				OLR	30	59 (26–75)	3/27	–	–	16 (53.33)	–	7.0 (5.2–12.0)	–	–	
Li 2019^[68]^	China	RM	2012–2017	LLR	32	–	–	–	–	–	–	–	–	–	7
				OLR	96	–	–	–	–	–	–	–	–	–	
Zeng 2019^[29]^	China	P	2017–2018	LLR	38	54.0 ± 10.6	33/5	23.3 ± 3.1	35/3/–	–	27 (71.1)	5.0 ± 2.7	–	28 (73.7)	5
				OLR	43	54.0 ± 10.0	36/7	23.8 ± 2.9	37/6/–	–	38 (88.4)	5.7 ± 2.5		24 (55.8)	
Yoon 2020^[61]^	Korea	RM	2007–2016	LLR	217	56.4 ± 9.7	170/47	–	–	145 (66.8)	185 (85.3)	2.8 ± 1.3	365 ± 1146	–	9
				OLR	434	56.9 ± 9.2	337/97	–	–	284 (65.4)	372 (85.7)	2.9 ± 1.3	273 ± 942	–	
Yi 2020^[63]^	China	RM	2008–2009	LLR	27	58.0 ± 12.1	20/7	–	–	18 (66.7)	21 (77.8)	5.1 ± 2.3	9 (1–1717)	25 (92.6)	8
				OLR	52	57.4 ± 10.8	43/9	–	–	34 (65.4)	42 (80.8)	5.3 ± 2.9	11 (1–1580)	52 (100)	
Dumronggittigule 2020^[62]^	Korea	RM	2003–2018	LLR	41	73 (71–79)	28/13	23.5 (21.8–25.4)	39/2	17 (41.5)	–	4 (2.5–5.7)	5 (3–19)	32 (78.0)	8
				OLR	41	73 (71–75)	35/6	22.8 (21.3–24.8)	37/4	24 (58.5)	–	4.1 (2.7–7.0)	7 (4–125)	36 (87.8)	
Chen 2020^[66]^	China	RM	2013–2018	LLR	64	71 (70–77)	41/23	20 (19–23)	–	–	–	2 (0.5–1.0)	–	–	6
				OLR	64	72 (70–76)	38/26	21 (18–27)	–		–	3 (0.4–0.9)	–	–	
Taesombat 2020^[65]^	Thailand	RM	2007–2013	LLR	27	61.5 ± 8.2	–	23.6 ± 4.9	27/–/–	23 (85.2)	15 (55.6)	3.8 ± 0.6	236 ± 564	–	7
				OLR	27	56.9 ± 9.5	–	23.5 ± 3.9	27/–/–	14 (51.9)	19 (70.4)	4.5 ± 1.9	107 ± 286	–	
Nomi 2020^[64]^	Japan	RM	2010–2017	LLR	155	78 (75–93)	89/66	22.5 (15.9–31.9)	–	–	–	2.8 (0.2–12)	–	–	8
				OLR	155	78 (75–87)	103/52	22.5 (14.7–33.2)	–	–	–	2.8 (0.2–15)	–	–	
Delvecchio 2021^[57]^	Italy	RM	2009–2019	LLR	38	74.3 (70–86)	29/9	26 (18–34)	37/1/–	–	10 (27)	4 (3–16)	700 (5–8904)	33 (87)	8
				OLR	84	75 (70–82)	61/23	26.7 (15–37)	82/2/–	–	16 (19)	7 (1.5–14)	830 (2–39 297)	68 (81)	
Troisi 2021^[59]^	Italy	RM	2002–2018	LLR	100	68 (27–84)	25/75	24 (18–41)	–/100/–	–	–	3 (0.8–14)	–	–	8
				OLR	100	69 (30–84)	22/73	24 (17–40)	–/100/–	–	–	3 (0.8–12)	–	–	
Navarro 2021^[56]^	Korea	RM	2013–2018	LLR	83	–	67/16	–	–	–	59 (71.1)	–	–	–	7
				OLR	83	–	69/14		–		65 (78.3)	–	–	–	
He 2021^[55]^	China	RM	2012–2018	LLR	26	56.1 ± 10.6	11/15	23.8 ± 3.0	23/3/–	16 (61.5)	–	7.5 ± 3.5	–	–	7
				OLR	78	52.0 ± 12.2	33/45	22.8 ± 2.7	70/8/–	45 (57.7)	–	7.6 ± 3.9	–	–	
Wong 2021^[52]^	China	RM	2008–2018	LLR	46	70 (65–85)	31/15	24.2 (17–31)	45/1/–	29 (63)	32 (69.6)	3.3 (1.2–7)	18 (2–517)	42 (91.3)	7
				OLR	92	71 (65–83)	62/30	23.5 (14–35)	92/–/–	60 (65.2)	68 (73.9)	4 (1–14)	8 (1–13 209)	78 (84.8)	
Gau 2021^[60]^	Taiwan	RM	2012–2019	LLR	73	60.4 ± 11.7	54/19	–	73/–/–	–	–	3.2 ± 1.5	–	–	9
				OLR	73	58.2 ± 11.1	60/13	–	73/–/–	–	–	3.2 ± 1.6	–	–	
Uemoto 2021^[51]^	Japan	RM	2011–2019	LLR	68	–	52/16	23.2 ± 3.4	–	19 (27.9)	14 (20.6)	3.4 ± 2.6	7 (1–25 265)	–	9
				OLR	68	–	52/16	23.2 ± 2.8	–	18 (26.5)	11 (16.2)	3.0 ± 2.3	10 (1–6816)	–	
Ho 2021^[53]^	China	RM	2000–2019	LLR	45	62 (57.5–68)	37/8	–	45/0/0	26 (57.8)	42 (93.3)	3.5 (2–5)	14 (4–358)	37 (82.2)	7
				OLR	90	62 (54.8–71)	72/18	–	73/7/0	58 (64.4)	72 (80)	4 (3–5)	22 (5–404)	70 (77.8)	
Lee 2021^[58]^	Korea	RM	2009–2018	LLR	58	57 (33–82)	34/24	24.0 (18.7–33.0)	52/6/–	35 (60.3)	53 (91.4)	2.4 (0.7–14)	13 (1–1675)	–	9
				OLR	58	59 (34–81)	35/23	23.4 (19.1–31.2)	53/5/–	39 (67.2)	54 (93.1)	2.6 (1.1–14.5)	15 (1–1580)	–	
Wen 2021^[54]^	China	RM	2015–2018	LLR	71	68 (66–72)	54/72	23.1 ± 3.2	–	25 (35.2)	28 (39.4)	5.5 (4.0–7.5)	7 (4–7)	50 (70.4)	8
				OLR	71	69 (66–72)	57/14	23.0 ± 3.0	–	27 (38.0)	24 (33.8)	6.0 (4.0–8.0)	8 (4–76)	53 (74.6)	
Peng 2022^[46]^	China	RM	2015–2018	LLR	72	54 (31–76)	59/13	22.9 (17.6–31.2)	69/3/–	52 (72.2)	54 (75.0)	5 (3.0–15.5)	–	0	8
				OLR	72	57 (21–76)	60/12	22.8 (17.6–32.4)	68/4/–	54 (75.0)	56 (77.7)	5.1 (1.5–11)	–	0	
Delvecchio 2024^[33]^	France	RM	2015–2020	LLR	219	75 (70–94)	158/61	26.7 (18–52)	187/32/–	95 (43)	53 (24)	3.5 (0.9–16)	530 (0–44 100)	196 (89)	8
				OLR	219	75 (70–89)	167/52	26.7 (15–41)	199/20/–	86 (39)	33 (15)	4 (0.7–15)	243 (2–9375)	190 (87)	
Monden 2022^[48]^	Japan	RM	2010–2021	LLR	75	75 (70–83)	53/22	23.1 (16.6–34.5)	71/4/–	–	15 (20.0)	2.4 (1–8.2)	8 (2–8900)	–	8
				OLR	75	75 (70–90)	51/24	23.0 (15.3–29.9)	72/3/–	–	14 (18.7)	2.1 (0.3–8)	10 (2–5359)	–	
Tian 2023^[40]^	China	RM	2009–2017	LLR	567	50 (44–59)	484/83	–	566/1/–	379 (66.8)	495 (87.3)	–	–	524 (92.4)	9
				OLR	567	50 (44–60)	501/66	–	566/1/–	382 (67.4)	502 (88.5)	–	–	520 (91.7)	
Nam 2023^[45]^	Korea	RM	2010–2020	LLR	49	58.2 ± 11.1		23.8 ± 3.0	–	–	39 (79.6)	5.5 ± 3.3	18 (5–232)	–	9
				OLR	49	60.4 ± 11.6		24.2 ± 3.3	–	–	39 (79.6)	5.5 ± 3.0	23 (5–494)	–	
Dumronggittigule 2023^[38]^	Korea	RM	2003–2018	LLR	70	60 (51–70)	60/10	24.0 (22.0–25.8)	66/4/–	30 (42.9)	–	6.0 (5.5–7.3)	17 (4–420)	54 (77.1)	8
				OLR	70	60 (49–67)	64/6	23.0 (21.6–24.9)	66/4/–	33 (47.1)	–	6.0 (5.3–8.0)	30 (4–1252)	63 (90.0)	
Yamasaki 2023^[41]^	Japan	RM	2010–2021	LLR	34	72 (67–75)	30/4	23.8 (22.0–26.3)	34/–/–	8 (24)	–	3.0 (2.4–3.8)	–	32 (94)	7
				OLR	34	73 (64–78)	30/4	24.4 (21.8–27.3)	34/–/–	6 (18)	–	3.4 (1.9–4.1)	–	33 (97)	
Zhang 2024^[36]^	China	RM	2010–2017	LLR	73	–	62/11	–	–	55 (75.3)	64 (87.7)	7.5 (6.0–9.0)	–	58 (79.5)	8
				OLR	73	–	63/10	–	–	55 (75.3)	61(83.6)	7.5 (6.0–10.5)	–	61 (83.6)	
Ding 2023^[42]^	China	RM	2014–2021	LLR	35	54.3 ± 12.8	28/7	–	–	17 (48.6)	30 (85.7)	6 (5.2–7.6)	18 (5–336)	–	8
				OLR	84	55.6 ± 9.8	71/13	–	–	49 (58.3)	68 (81)	6.9 (5.4–8.6)	179 (14–1210)	–	
Park 2023^[39]^	Korea	RM	2008–2017	LLR	25	60.6 ± 10.2	20/5	24.5 ± 3.5	–	10 (40.0)	16 (64.0)	4.9 ± 2.8	673 ± 1750	21 (84)	8
				OLR	25	57.4 ± 12.6	20/5	23.8 ± 3.2	–	13 (52.0)	14 (56.0)	4.6 ± 2.8	1813 ± 66 081	23 (92)	
Ng 2023^[32]^	Hong Kong	RM	2010–2020	LLR	65	63 (31–85)	55/10	–	64/1/–	26 (40.0)	51 (78.5)	4.5 (0.7–13)	29 (1–15 863)	50 (76.9)	8
				OLR	130	62 (36–83)	107/23	–	127/3/–	56 (43.1)	103 (79.2)	3.7 (0.4–13)	12 (1–394 685)	95 (73.1)	
Watanabe 2024^[34]^	Japan	RM	2007–2022	LLR	46	74 (69–79)	39/7	25.4 ± 4.8	45/1/–	16 (35)	–	3 (2–6)	4 (3–13)	–	8
				OLR	46	72 (68–77)	39/7	25.4 ± 3.7	46/–/–	17 (37)	–	4 (2–6)	8 (3–26)	–	
Sakai 2024^[31]^	Japan	RM	2010–2024	LLR	100	72 (43–87)	75/25	23.6 (15.7–37)	100/–/–	18 (18)	16 (16.0)	2.8 (1.2–10)	5 (1–4162)	81 (81)	8
				OLR	100	72 (53–87)	70/30	23.0 (16.1–38)	100/–/–	21 (21)	15 (15.0)	2.5 (0.5–8.5)	6 (1–2200)	82 (82)	

Data are shown as mean ± standard deviation, median (range), or n (%) BMI, body mass index; CTP, Child–Turcotte–Pugh; HBV, hepatitis B virus; AFP, alpha-Fetoprotein; NOS, Newcastle–Ottawa Scale; ROB, risk of bias; RM, retrospective-matched; P, prospective; RCT, randomized controlled trials; LLR, laparoscopic liver resection; RLR, robotic liver resection; OLR, open liver resection; SC, some concerns.

Data are shown as mean ± standard deviation, median (range), or *n* (%)

BMI, body mass index; CTP, Child–Turcotte–Pugh; HBV, hepatitis B virus; AFP, alpha-Fetoprotein; NOS, Newcastle–Ottawa Scale; ROB, risk of bias; RM, retrospective-matched; P, prospective; RCT, randomized controlled trials; LLR, laparoscopic liver resection; RLR, robotic liver resection; OLR, open liver resection; SC, some concerns.

### Quality of included studies

Study quality was assessed using the Cochrane risk-of-bias version 2 tool for RCTs and the Newcastle–Ottawa Scale for prospective and retrospective matched studies. Except for four, most studies were classified as high quality. A summary of these assessments is shown in Supplemental Digital Content Table S3, available at http://links.lww.com/JS9/F301.

### Meta-analysis of two-arm studies

#### Laparoscopic vs. robot liver resection

Table 2 summarizes the comparative outcomes from pooled analyses of 5 studies (1071 patients)^[1,9,35,43,49]^, with detailed results shown in Supplemental Digital Content Figures S1–S3, available at http://links.lww.com/JS9/F302.

**Table 2:** League table showing indirect comparisons among surgical approaches

Outcomes									
Operative	**Operative time^a^**					**Estimated blood loss**^a^	
	Laparoscopic					Laparoscopic			
	10.62 [0.81–20.43]		Open			4.38 [−5.16 to 13.91]		Open	
	4.22 [−30.54 to 22.11]		−14.84 [−40.31 to 10.63]		Robot	94.17 [69.15–119.20]		89.8 [65.93–113.66]	Robot
	**RBC transfusion^b^**					**Pringle maneuver b**			
	Laparoscopic					Laparoscopic			
	0.62 [0.52–0.74]		Open			0.65 [0.47–0.89]		Open	
	0.67 [0.43–1.05]		1.07 [0.69–1.68]		Robot	1.16 [0.55–2.45]		1.79 [0.88–3.61]	Robot
	**Pringle time^a^**					**R1 resection^b^**			
	Laparoscopic					Laparoscopic			
	6.54 [0.42–12.67]		Open			0.70 [0.52–0.93]		Open	
	3.62 [−10.36 to 17.60]		−2.92 [−16.07 to 10.22]		Robot	0.66 [0.20–2.16]		0.95 [0.29–3.08]	Robot
Postoperative	**Overall complication^b^**					**Major complication^b^**			
	Laparoscopic					Laparoscopic			
	0.52 [0.45–0.61]		Open			0.46 [0.39–0.56]		Open	
	1.07 [0.68–1.68]		2.04 [1.31–3.18]		Robot	1.49 [0.86–2.61]		3.22 [1.88–5.51]	Robot
	**90 days mortality^b^**					**Hospital stays^a^**			
	Laparoscopic					Laparoscopic			
	0.60 [0.38–0.95]		Open			−4.48 [−5.11 to −3.85]		Open	
	0.75 [0.36–1.56]		1.25 [0.60–2.60]		Robot	−1.44 [−3.00 to 0.13]		3.04 [1.51–4.57]	Robot
Long-term	**1-Year OS^c^**					**1-Year RFS^c^**			
	Laparoscopic					Laparoscopic			
	0.76 [0.64–0.89]		Open			0.94 [0.84–1.05]		Open	
	1.08 [0.74–1.56]		1.42 [1.01–2.01]		Robot	1.07 [0.81–1.41]		1.13 [0.87–1.49]	Robot
	**3-Year OS^c^**					**3-Year RFS^c^**			
	Laparoscopic					Laparoscopic			
	0.96 [0.86–1.06]		Open			0.95 [0.89–1.03]		Open	
	1.07 [0.84–1.35]		1.11 [0.90–1.38]		Robot	1.05 [0.87–1.26]		1.10 [0.92–1.31]	Robot
	**5-Year OS^c^**					**5-Year RFS^c^**			
	Laparoscopic					Laparoscopic			
	0.96 [0.86–1.07]		Open			0.97 [0.89–1.04]		Open	
	0.97 [0.78–1.22]		1.02 [0.82–1.25]		Robot	1.03 [0.86–1.25]		1.07 [0.89–1.28]	Robot

Open liver resection served as the reference treatment with an ORs and HRs of 1.00 or SMD of 0.00.

^a^ presented as standardized mean difference [95% confidence interval].

^b^ presented as odd ratio [95% confidence interval].

^c^ presented as hazard ratio [95% confidence interval].

RBC, red blood cell; OS, overall survival; RFS, recurrence-free survival.

*Operative outcomes*: LRL demonstrated a significant reduction in operative time (SMD: −0.31; 95% CI: −0.56 to 0.07;*P* = 0.01), while no significant differences were observed between LRL and RLR in terms of estimated blood loss, red blood cell transfusion requirement, and R1 resection rate.

*Postoperative outcomes*: RLR was associated with a significantly lower incidence of overall complications compared to LRL (OR: 2.23; 95% CI: 1.30–3.82; *P* = 0.004), while no significant differences were observed between LRL and RLR in major complications, 90-day mortality, and length of hospital stay.

*Long-term outcomes*: No significant differences were observed between LRL and RLR in 1-, 3-, and 5-year OS and RFS.

#### Robot vs. open liver resection

Table 2 summarizes the this meta-analysis outcomes from 7 studies (1615 patients)^[1,36,37,43,47,50,75]^, with detailed results shown in Supplemental Digital Content Figures S4–S6, available at http://links.lww.com/JS9/F302.

*Operative outcomes*: Pringle maneuver application was significantly less in RLR compared to OLR (OR: 1.17; 95% CI: 1.31–2.22; *P*<0.001), while no significant differences were found between OLR and RLR regarding operative time, estimated blood loss, red blood cell transfusion, Pringle time, and R1 resection rate.

*Postoperative outcomes*: RLR was associated with a significantly reduced rate of major complications (OR: 2.64; 95% CI: 1.47–4.77; *P* = 0.001) and shorter hospital stays (SMD: 2.64; 95% CI: 1.47–4.77; *P* = 0.001) compared to OLR, while no significant differences were observed between OLR and RLR in overall complication rates or 90-day mortality.

*Long-term outcomes*: There were no significant differences between LRL and RLR in terms of OS and RFS except for 1-year OS, which was significantly better in RLR (HR: 1.55; 95% CI: 1.07–2.24; *P* = 0.02).

#### Laparoscopic vs. open liver resection

Table 2 summarizes the this meta-analysis outcomes from 61 studies (10 845 patients)^[1,28–38,40,42,44–69,71–74,76–85,87–91,93,94]^, with detailed results shown in Supplemental Digital Content Figures S7–S9, available at http://links.lww.com/JS9/F302.

*Operative outcomes*: OLR significantly reduced operative time compared to LLR (SMD: 0.28; 95% CI: 0.09–0.47; *P* = 0.004), whereas LLR demonstrated significant advantages in reducing estimated blood loss (SMD: −0.53; 95% CI: −0.75 to −0.31; *P* < 0.001), red blood cell transfusion (OR: 0.63; 95% CI: 0.52–0.75; *P*<0.001), Pringle maneuver application (OR: 0.62; 95% CI: 0.43–0.89; *P* = 0.01), and R1 resection (OR: 0.69; 95% CI: 0.51–0.92; *P* = 0.01), with no significant difference in Pringle time between the two approaches.

*Postoperative outcomes*: LRL demonstrated superior postoperative outcomes compared to OLR, including reduced rates of overall complications (OR: 0.51; 95% CI: 0.44–0.58; *P*<0.001), major complication (OR: 0.46; 95% CI: 0.38–0.55; *P*<0.001), 90-day mortality (OR: 0.45; 95% CI: 0.25–0.82; *P* = 0.008), and shorter hospital stays (SMD: −0.84; 95% CI: −1.01 to −0.68; *P*<0.001).

*Long-term outcomes*: No significant differences were observed between LLR and OLR in terms of OS and RFS, except for 1-year OS, which was significantly improved with LLR (HR: 0.77; 95% CI: 0.65–0.91; *P* = 0.002).

#### Subgroup analysis for major and minor resection

Subgroup analysis was performed by resection extent, defined as major (≥3 segments) or minor (≤2 segments). Outcome data by resection type were extracted from individual studies. Since only one study including robotic surgery met the eligibility criteria^[47]^, the subgroup meta-analysis was performed only for comparisons between LLR and OLR with 20 studies^[38–40,55–57,63,66,67,70–73,76,77,81,85,90,91,93]^.

In both major and minor resection subgroups, pooled analyses showed no significant differences between LLR and OLR in operative, postoperative, or long-term outcomes (Supplemental Digital Content Figures S10–S12, available at http://links.lww.com/JS9/F302). These results are summarized in Supplemental Digital Content Table S4, available at http://links.lww.com/JS9/F301.

### Network meta-analysis

A frequentist framework was used to rank treatments using random effects *P*-scores, with a score of 1 indicating the theoretically optimal treatment and 0 indicating the least favorable. In the analyses, OLR was designated as the reference treatment (OR/HR: 1.00; SMD: 0.00).

**Figure 2.**
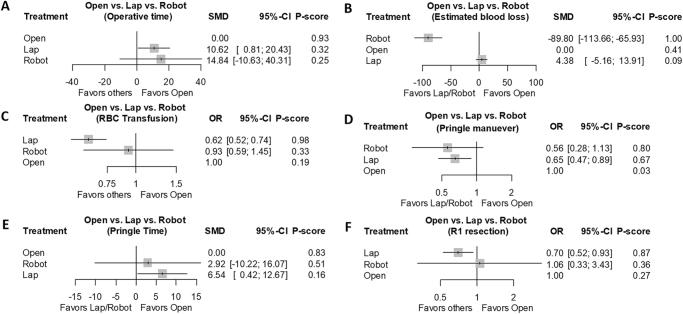
Network meta-analysis of operative outcomes. (A) Operative time, (B) estimated blood loss, (C) RBC transfusion, (D) Pringle maneuver, (E) Pringle time, and (F) R1 resection.

*Operative outcomes*: OLR showed a shorter operative time than LLR (SMD: 10.62; 95% CI: 0.81–20.43). RLR reduced estimated blood loss compared to both LLR (SMD: 94.17; 95% CI: 69.15–119.20) and OLR (SMD: 1.79; 95% CI: 0.88–3.61). LLR required fewer red blood cell transfusions (OR: 0.62; 95% CI: 0.52–0.74) and less Pringle maneuver application (OR: 0.65; 95% CI: 0.47–0.89) than OLR. OLR reduced Pringle time compared to LLR (SMD: 6.54; 95% CI: 0.42–12.67). R1 resection was less frequent in LLR compared to OLR (OR: 0.70; 95% CI: 0.52–0.93). Detailed results are presented in Table 3, with rankograms illustrating rankings as follows: operative time (rank-order/*P*-score: OLR-LLR-RLR/0.93-0.32-0.25), estimated blood loss (RLR-OLR-LLR/1.00-0.41-0.09), red blood cell transfusion (LLR-RLR-OLR/0.98-0.33-0.19), Pringle maneuver (RLR-LLR-OLR/0.80-0.67-0.03), Pringle time (OLR-RLR-LLR/0.83-0.51-0.16), and R1 resection (LLR-RLR-OLR/0.87-0.36-0.27) (Fig. 2A-F).

**Table 3:** Outcomes of meta-analyses in two-arm studies

Groups/variables	Number of studies	Pooled results			Heterogeneity	
		*N*	Estimate [95% CI]	*P*	*I*^2^ (%)	*P*_H_
Laparoscopic vs. robot						
Operative time	3^[1,35,43]^	481	SMD: −0.31 [−0.56 to −0.07]	0.01	21	0.28
Estimated blood loss	2^[1,35]^	453	SMD: 0.02 [−0.18 to 0.22]	0.83	0	0.74
RBC transfusion	4^[1,9,35,43]^	567	OR: 0.56 [0.31–1.00]	0.05	0	0.73
Pringle maneuver	1^[1]^	112	–	–	–	–
Pringle time	1^[1]^	112	–	–	–	–
R1 resection	2^[1,43]^	140	OR: 0.73 [0.07–8.01]	0.79	34	0.22
Overall complication	4^[1,9,35,43]^	567	OR: 2.23 [1.30–3.82]	0.004	0	0.58
Major complication	3^[1,9,43]^	226	OR: 2.36 [0.53–10.55]	0.26	33	0.22
90-day mortality	3^[1,9,35]^	539	OR: 1.16 [0.35–3.83]	0.81	15	0.28
Hospital stays	4^[1,9,35,43]^	567	SMD: 0.06 [−0.72 to 0.83]	0.88	93	<0.001
1-year OS	2^[1,35]^	453	HR: 0.65 [0.26–1.61]	0.35	0	0.80
3-year OS	2^[1,35]^	453	HR: 0.78 [0.52–1.71]	0.23	0	0.52
5-year OS	2^[1,35]^	453	HR: 0.78 [0.52–1.71]	0.23	0	0.52
1-year RFS	2^[1,35]^	453	HR: 1.16 [0.43–3.07]	0.77	80	0.03
3-year RFS	2^[1,35]^	453	HR: 1.20 [0.70–2.04]	0.51	56	0.13
5-year RFS	2^[1,35]^	453	HR: 1.26 [0.63–2.55]	0.51	81	0.02
Open vs. robot						
Operative time	6^[1,36,37,43,50,75]^	1551	SMD: −0.23 [−0.89 to 0.42]	0.48	97	<0.001
Estimated blood loss	5^[1,36,37,50,75]^	1509	SMD: 0.57 [−0.95 to 2.08]	0.46	99	<0.001
RBC transfusion	5^[1,37,43,50,75]^	806	OR: 1.22 [0.66–2.25]	0.52	19	0.30
Pringle maneuver	3^[1,36,50]^	1135	OR: 1.71 [1.31–2.22]	<0.001	0	0.98
Pringle time	3^[1,36,50]^	1135	SMD: −0.34 [−0.96 to 0.29]	0.29	95	<0.001
R1 resection	4^[1,37,43,75]^	528	OR: 0.77 [0.19–3.14]	0.72	0	0.65
Overall complication	5^[1,37,43,50,75]^	806	OR: 1.54 [0.76–3.10]	0.23	63	0.03
Major complication	6^[1,36,37,43,50,75]^	1551	OR: 2.64 [1.47–4.77]	0.001	0	0.71
90-day mortality	2^[1,50]^	390	OR: 0.85 [0.37–1.94]	0.70	0	0.81
Hospital stays	6^[1,36,37,43,50,75]^	1551	SMD: 2.64 [1.47–4.77]	0.001	0	0.71
1-year OS	6^[1,36,37,47,50,75]^	1535	HR: 1.55 [1.07–2.24]	0.02	0	0.57
3-year OS	6^[1,36,37,47,50,75]^	1535	HR: 1.17 [0.96–1.44]	0.13	5	0.39
5-year OS	4^[1,36,37,50]^	1347	HR: 1.07 [0.85–1.35]	0.55	34	0.21
1-year RFS	4^[1,36,50,75]^	1297	HR: 1.10 [0.75–1.60]	0.63	67	0.03
3-year RFS	4^[1,36,50,75]^	1297	HR: 1.05 [0.86–1.30]	0.62	46	0.13
5-year RFS	3^[1,36,50]^	1135	HR: 0.99 [0.86–1.14]	0.93	14	0.31
Laparoscopic vs. open						
Operative time	50^[28–34,38–41,43,45,46,48,51,53–58,60–62,65–67,70–74,77–86,88–94]^	8818	SMD: 0.28 [0.09–0.47]	0.004	94	<0.001
Estimated blood loss	46^[28–30,38–41,45,46,48,51–56,60–67,70,71,73,74,76,77,79–81,83–91,93,94]^	8124	SMD: −0.53 [−0.75 to −0.31]	<0.001	95	<0.001
RBC transfusion	46^[28,30–34,38–41,43–45,51–55,57,58,61–67,69–74,76,77,79,80,82–84,88,89,91–94]^	8593	OR: 0.63 [0.52–0.75]	<0.001	23	0.09
Pringle maneuver	22^[30,32,33,38,45,53,57,59–61,64,65,67,69,72,76,79,84,90–92,94]^	3806	OR: 0.62 [0.43–0.89]	0.01	77	<0.001
Pringle time	17^[30,32,34,38,41,54,59,60,64,71,76,79,84,88,90,91,93]^	2256	SMD: 0.38 [−0.07 to 0.83]	0.10	96	<0.001
R1 resection	39^[28,31–34,38–41,43,46,48,51–53,55,57–60,62,64–66,71,73,74,76,79–85,87,88,92,93]^	7639	OR: 0.69 [0.51–0.92]	0.01	12	0.27
Overall complication	54^[28,31–34,38–41,43,46,48,51–53,55,57,58,60,62,64–66,71,73,74,76,79–85,87,88,92,93]^	9399	OR: 0.51 [0.44–0.58]	<0.001	23	0.07
Major complication	49^[28,30–34,38–46,51,53,54,57–62,64–69,71–74,77–85,87,89,91–94]^	8515	OR: 0.46 [0.38–0.55]	<0.001	0	0.79
90-day mortality	27^[28,30–34,38–46,51,53,54,57–62,64–69,71–74,77–85,87,89,91–94]^	5167	OR: 0.45 [0.25–0.82]	0.008	0	0.96
Hospital stays	54^[29–34,38–46,48,51–67,70–72,74,76–94]^	9378	SMD: −0.84 [−1.01 to −0.68]	<0.001	92	<0.001
1-year OS	49^[30–34,38–40,42,44–46,52–63,65–70,72–74,76–84,86,87,89–91,94]^	9231	HR: 0.77 [0.65–0.91]	0.002	0	0.69
3-year OS	49^[30–34,38–40,42,44–46,52–63,65–70,72–74,76–84,86,87,89–91,94]^	9231	HR: 0.98 [0.88–1.10]	0.77	9	0.30
5-year OS	36^[31–34,38–40,44,45,52,53,55–62,65,66,69,72–74,77,80,81,83,84,86–91,94]^	7706	HR: 0.98 [0.87–1.09]	0.70	25	0.09
1-year RFS	49^[28,30–34,38–40,44–46,52–63,65–70,72–74,76–84,86,87,89–92,94]^	9217	HR: 0.94 [0.84–1.05]	0.27	30	0.03
3-year RFS	49^[28,30–34,38–40,44–46,52–63,65–70,72–74,76–84,86,87,89–92,94]^	9217	HR: 0.94 [0.88–1.01]	0.10	22	0.09
5-year RFS	38^[31–34,38–40,44,45,52,53,55–62,65,66,69,72–74,77,80,81,83,84,86,87,89–92,94]^	7761	HR: 0.95 [0.89–1.03]	0.20	27	0.07

Random effect model was applied for all outcomes. OR, odds ratio; HR, hazard ratio; SMD, standardized mean difference; CI, confidence interval; RBC, red blood cell; OS, overall survival; RFS, recurrence-free survival.

Random effect model was applied for all outcomes.

OR, odds ratio; HR, hazard ratio; SMD, standardized mean difference; CI, confidence interval; RBC, red blood cell; OS, overall survival; RFS, recurrence-free survival.

**Figure 3.**
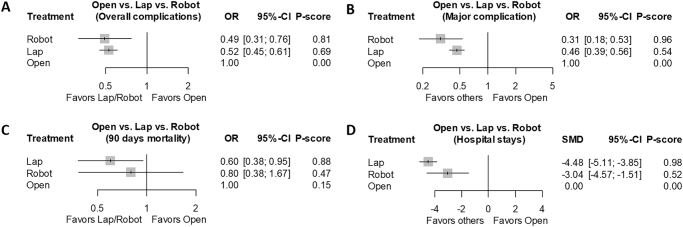
Network meta-analysis of postoperative outcomes. (A) Overall complication, (B) major complication, (C) 90-day mortality, and (D) hospital stays.

*Postoperative outcomes*: Both minimally invasive approaches showed better outcomes than OLR. Specifically, RLR (OR: 2.04; 95% CI: 1.31–3.18) and LLR (OR: 0.52; 95% CI: 0.45–0.61) reduced overall complications and also lowered major complications (RLR: OR 3.22; 95% CI: 1.51–4.57; LLR: OR 0.46; 95% CI: 0.39–0.56). LLR additionally provided a 90-day mortality benefit (OR: 0.60; 95% CI: 0.38–0.95). Both LLR (SMD: −4.48; 95% CI: −5.11 to −3.85) and RLR (SMD: 3.04; 95% CI: 1.51–4.57) showed shorter hospital stays compared to OLR. Detailed results are summarized in Table 3, with rankograms displaying rankings: overall complication (rank-order/*P*-score: RLR-LLR-OLR/0.81-0.69-0.00), major complication (RLR-LLR-OLR/0.96-0.54-0.00), 90-day mortality (LLR-RLR-OLR/0.88-0.47-0.15), and hospital stays (LLR-RLR-OLR/0.98-0.52-0.00) (Fig. 3A–D).

**Figure 4.**
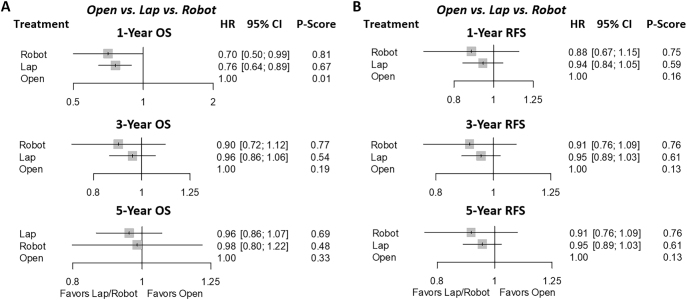
Network meta-analysis of long-term outcomes. (A) 1-, 3-, and 5-year overall survival and (B) 1-, 3-, and 5-recurrence-free survival.

*Long-term outcomes*: For 1-year OS, RLR (HR: 1.42; 95% CI: 1.01–1.25) and LLR (HR: 0.76; 95% CI: 0.64–0.89) showed a survival benefit compared with OLR. No significant differences were seen among the three approaches for 3- and 5-year OS or for 1-, 3-, and 5-year RFS. Detailed results are showed in Table 3, with rankograms illustrating rankings as follows: 1-year OS (rank-order/*P*-score: RLR-LLR-OLR/0.81-0.67-0.01), 3-year OS (RLR-LLR-OLR/0.77-0.54-0.19), and 5-year OS (RLR-LLR-OLR/0.69-0.48-0.33) and 1-year RFS (RLR-LLR-OLR/0.75-0.59-0.16), 3-year RFS (RLR-LLR-OLR/0.76-0.61-0.13), and 5-year OS (RLR-LLR-OLR/0.76-0.61-0.13) (Fig. 4A–B).

#### Sensitivity test and publication bias

A sensitivity analysis was performed by excluding four studies^[28,29,60,89]^ that did not meet high-quality criteria based on Cochrane risk-of-bias tool for randomized trials version 2 and Newcastle–Ottawa Scale. In the analysis of 65 high-quality studies, no significant differences were observed in operative, postoperative, or long-term outcomes compared to the meta-analyses with two-arm studies (Supplemental Digital Content Table S5, available at http://links.lww.com/JS9/F301) and network meta-analysis (Supplemental Digital Content Table S6, available at http://links.lww.com/JS9/F301). The symmetrical pattern in the comparison-adjusted funnel plot for primary outcomes, 5-year OS, with the majority of studies falling within the 95% CI, suggested no significant publication bias in this analysis (Supplemental Digital Content Figure S13, available at http://links.lww.com/JS9/F302).

## DISCUSSION

Minimally invasive liver surgery, including laparoscopic, single-incision laparoscopic, and robotic-assisted hepatectomies, has become increasingly preferred over OLR for HCC due to perioperative advantages such as shorter hospital stay, lower transfusion rates, fewer complications, reduced 90-day mortality, and faster recovery^[12,13]^. Minimally invasive and open procedures have demonstrated comparable long-term OS and RFS in HCC patients, including those with cirrhosis or metabolic syndrome^[55,95,96]^. For early-stage HCC and elderly patients, minimally invasive surgery provides better short-term outcomes and comparable long-term outcomes to OLR^[6,95]^. Although minimally invasive liver surgery is technically challenging in patients with advanced cirrhosis, portal hypertension, or tumors in difficult segments, it can be performed safely in high-volume centers by experienced surgeons, but the higher rate of positive margins requires further study^[97]^.

There is still a lack of well-designed comparative studies between RLR and LLR specifically for HCC, although RLR has rapidly emerged as a new trend. Most RLR studies have evaluated short-term outcomes in benign or mixed tumor cases rather than focusing on HCC^[13]^. Recent meta-analyses have presented that RLR achieves short-term outcomes comparable to those of LLR, with similar rates of blood loss, morbidity, and mortality; however, RLR is frequently associated with longer operative durations^[10–13]^. For HCC patients with cirrhosis and other comorbidities, both RLR and LLR are feasible and safe, providing favorable short-term outcomes, while long-term outcomes are generally comparable^[1,75]^. Both techniques effectively preserve oncological integrity, showing comparable rates of R0 resection margins^[14]^. Data on conversion rates between laparoscopic and robotic hepatectomy for HCC remain limited and inconsistent. Duong *et al*^[49]^ reported higher rates with robotic resection in an unmatched cohort (8.1% vs. 3.9%), and Zhu *et al*^[1]^ found similar results in a matched cohort (12.5% vs. 14.3%). In contrast, O’Connell *et al*^[43]^ observed a higher conversion rate with laparoscopic resection (21% vs. 0%) in a small series. Overall, evidence is insufficient and lacks consistency. Although robotic liver surgery may facilitate suturing and management of major vascular injuries^[11–13,98,99]^, larger studies are required to better define its impact on conversion. Expert opinion supports RLR as a safe and effective minimally invasive approach, providing advantages such as enhanced dexterity, three-dimensional visualization, and improved ergonomics for complex resections^[5]^. Compared with laparoscopic and open surgery, RLR offers comparable safety and oncologic outcomes, with potential benefits of reduced blood loss and faster recovery in selected cases^[5,92]^. Although limited to only two studies, our two-arm meta-analysis showed no significant difference in blood loss between RLR and LLR. Importantly, the robotic platform appears to shorten the learning curve, with competency, proficiency, and mastery reported after approximately 23 and 63 cases, respectively^[98]^. Furthermore, RLR has been associated with excellent perioperative outcomes, and its indications have gradually expanded to major liver resections. With ongoing studies and future technological advances, robotic platform holds significant potential to further advance minimally invasive hepatobiliary surgery^[5,86,92]^.

Only one previous meta-analysis reported long-term outcomes comparing RLR with LLR or OLR in HCC, but it included only eight studies, most of which were unmatched and retrospective, limiting the reliability of the pooled results^[99]^. Therefore, this study updated the recent evidence using well-designed studies and provided pooled results for long-term outcomes of RLR, LLR, and OLR, as well as perioperative outcomes. Specifically, we conducted a network meta-analysis comparing long-term and perioperative outcomes of RLR, LLR, and OLR. The results clarify the relative performance of RLR and LLR and underscore the advantages of minimally invasive procedures over open approach in HCC, providing ranking data to guide treatment decisions in centers where RLR experience is still limited.

This study also included direct two-arm comparisons. LLR showed shorter operative times but higher overall complication rates than RLR, whereas RLR had lower Pringle maneuver use and fewer major complications than OLR. Compared with OLR, LLR was associated with longer operative times but reduced blood loss, red blood cell transfusion, Pringle maneuver use, R1 resection rate, overall and major complications, 90-day mortality, and shorter hospital stays. For long-term outcomes, no significant differences were observed between RLR and LLR, but both showed a 1-year OS benefit over OLR, likely reflecting higher postoperative mortality in OLR. Although this study analyzed only matched or prospective cohorts, potential selection bias remains, as OLR patients may have more complex tumors or require major resections. Overall, RLR had longer operative times than LLR but showed no significant inferiority to either LLR or OLR in perioperative or long-term outcomes. In the network meta-analysis, RLR demonstrated significantly lower blood loss than both OLR and LLR, with no disadvantage in operative time, red blood cell transfusion, Pringle maneuver use or duration, or R1 resection rates. RLR also had complication rates and hospital stay comparable to LLR, while offering notable benefits over OLR. No significant differences in 90-day mortality were found among the three groups. For long-term outcomes, RLR was not superior to LLR or OLR in OS or RFS, although it generally ranked higher with a greater *P*-score.

Subgroup analyses according to the extent of resection showed no significant differences in operative, postoperative, or long-term outcomes between LLR and OLR for either major (≥3 segments) or minor (≤2 segments) hepatectomy. However, because only one study including robotic resection met the eligibility criteria, a three-arm subgroup network meta-analysis could not be performed. These findings highlight the need for future studies comparing RLR with LLR and OLR that report outcomes stratified by the extent of resection to enable more comprehensive evaluation.

Heterogeneity was assessed using the inconsistency statistic (*I*^2^), classified as low, moderate, or high at thresholds of 25%, 50%, and 75%, respectively. Although not universally applicable, this categorization is widely used in meta-analyses; generally, a fixed-effects model is applied for low-to-moderate heterogeneity, whereas a random-effects model is preferred for high heterogeneity. It is important to note that when using a random-effects model, the included studies conducted under diverse conditions such as variations in surgical techniques, expertise, experience levels, and learning curves in MILS may not share a common effect size^[26]^. Given the variability in study settings and designs, we conducted a pooled analysis using a random-effects model. In comparative studies of MILS and OLR, however, there is a high risk of selection bias, as the minimally invasive surgery group often includes patients with more favorable operative conditions such as lower tumor burden, optimal tumor location, and higher resectability, particularly during the initial learning curve. To improve the reliability of our meta-analysis, we excluded retrospective unmatched studies and included only RCT, prospective studies, and retrospective-matched studies. Although this approach cannot completely eliminate potential selection bias, it provides the most reliable results based on the available evidence.

This meta-analysis has several limitations. First, HRs for OS and RFS were reconstructed from Kaplan–Meier curves rather than raw data, which may introduce inaccuracies^[24]^, although two reviewers independently verified these values. Second, only a few studies have directly compared short-term and long-term outcomes between RLR and LLR for HCC. To address this limitation, we conducted a network meta-analysis incorporating all available comparisons (RLR vs. LLR, RLR vs. OLR, and LLR vs. OLR) to maximize sample size and enable indirect comparisons. Nevertheless, well-designed studies focusing specifically on RLR versus LLR for HCC are still required to provide more reliable evidence. Finally, because of the limited number of RLR studies, subgroup analyses by tumor characteristics or resection type could not be performed.

This network meta-analysis indicates that RLR is a feasible option for HCC, showing perioperative and long-term outcomes comparable to LLR and clear advantages over OLR in postoperative morbidity and hospital stay. Nevertheless, the limited number of RLR cases, particularly for long-term outcomes, highlights the need for further studies to confirm its efficacy in HCC.

## Data Availability

Data for this network meta-analysis were extracted from literature available in a public electronic database. Upon request, the authors will provide these data to qualified researcher.

## Research registration unique identifying number (UIN)

This study was registered in ResearchRegistry.com (research registry1915).
